# The Interactive Complex between Cytomegalovirus Kinase vCDK/pUL97 and Host Factors CDK7–Cyclin H Determines Individual Patterns of Transcription in Infected Cells

**DOI:** 10.3390/ijms242417421

**Published:** 2023-12-13

**Authors:** Martin Schütz, Arne Cordsmeier, Christina Wangen, Anselm H. C. Horn, Emanuel Wyler, Armin Ensser, Heinrich Sticht, Manfred Marschall

**Affiliations:** 1 Institute for Clinical and Molecular Virology, Friedrich-Alexander University of Erlangen-Nürnberg (FAU), 91054 Erlangen, Germany; 2 Division of Bioinformatics, Institute of Biochemistry, Friedrich-Alexander University of Erlangen-Nürnberg (FAU), 91054 Erlangen, Germany; 3 Max-Delbrück-Center for Molecular Medicine (MDC), 13125 Berlin, Germany

**Keywords:** human cytomegalovirus, cyclin-dependent kinases (CDKs), viral CDK ortholog (vCDK/pUL97), vCDK/pUL97–cyclin binding, functional complexation with host CDK7, impact on RNA polymerase (RNAP II) in infected cells, first vCDK/pUL97-specific transcriptomic analysis

## Abstract

The infection of human cytomegalovirus (HCMV) is strongly determined by the host–cell interaction in a way that the efficiency of HCMV lytic replication is dependent on the regulatory interplay between viral and cellular proteins. In particular, the activities of protein kinases, such as cyclin-dependent kinases (CDKs) and the viral CDK ortholog (vCDK/pUL97), play an important role in both viral reproduction and virus–host interaction. Very recently, we reported on the complexes formed between vCDK/pUL97, human cyclin H, and CDK7. Major hallmarks of this interplay are the interaction between cyclin H and vCDK/pUL97, which is consistently detectable across various conditions and host cell types of infection, the decrease or increase in pUL97 kinase activity resulting from cyclin H knock-down or elevated levels, respectively, and significant trans-stimulation of human CDK7 activity by pUL97 in vitro. Due to the fact that even a ternary complex of vCDK/pUL97–cyclin H–CDK7 can be detected by coimmunoprecipitation and visualized by bioinformatic structural modeling, we postulated a putative impact of the respective kinase activities on the patterns of transcription in HCMV-infected cells. Here, we undertook a first vCDK/pUL97-specific transcriptomic analysis, which combined conditions of fully lytic HCMV replication with those under specific vCDK/pUL97 or CDK7 drug-mediated inhibition or transient cyclin H knockout. The novel results were further strengthened using bioinformatic modeling of the involved multi-protein complexes. Our data underline the importance of these kinase activities for the C-terminal domain (CTD) phosphorylation-driven activation of host RNA polymerase in HCMV-infected cells. The impact of the individual experimental conditions on differentially expressed gene profiles is described in detail and discussed.

## 1. Introduction

Human cytomegalovirus (HCMV) is a major worldwide-distributed human pathogen and represents the prototype of β-herpesviruses. HCMV infection establishes a life-long latency in the infected host. Phases of viral reactivation and the possibility of secondary infections can raise medical issues that have not been solved until today. While in immunocompetent individuals, HCMV may remain asymptomatic, it can induce severe symptoms and even life-threatening disease in immunosuppressed patients, such as transplant recipients and cancer or AIDS patients [1,2,3]. Most importantly, HCMV infection in the immunonaïve host, i.e., unborns and neonates, can lead to serious congenital HCMV infection (cCMV) that may occur during pregnancy [4,5,6]. Due to the exceptionally high rate of mother-to-child transmission, cCMV represents the most serious risk of obtaining developmental defects or cytomegalovirus inclusion disease [7,8,9]. In general, HCMV-related pathogenesis directly correlates with the efficiency of viral replication in affected organs and tissues. As a distinct virus-supportive parameter, the role of regulatory host factors is crucial for the productiveness and pathogenesis of HCMV infection in particular, based on the interactive virus–host protein complexes and their regulatory consequences [10,11,12]. In this regard, it should be emphasized that HCMV replication interferes with the cellular cyclin-dependent kinase (CDK)–cyclin machinery in a multifaceted fashion. Of specific interest for this study is the complex consisting of CDK7, cyclin H and MAT, also called the cyclin-activating kinase (CAK) complex [13]. This heterotrimeric complex is responsible for activating multiple CDKs via T-loop phosphorylation and plays an integral role in transcription as a subunit of the general transcription factor IIH [14,15,16]. Of note, the viral CDK ortholog, namely, vCDK/pUL97, which combines structural and functional properties of host CDKs [10,17,18,19,20], directly interacts with cyclins and CDKs in both physical contacts and regulatory processes [10,21]. Viral pUL97 is considered a multiple-cyclin-binding kinase, for which the interaction with cyclin H and CDK7 has particular relevance as a determinant of the HCMV replication efficiency [11]. In this regard, it is important that the minimal binding regions responsible for pUL97–cyclin H interaction maps in the poorly structured N-terminal amino acid region 231–280. Recent investigations provided evidence that the vCDK/pUL97 kinase activity is coregulated through cyclin H binding and that the interaction of this complex with CDK7 can lead to its trans-stimulation (at least when measured in activity assays in vitro). Specifically, this finding prompted us to address the question of whether vCDK/pUL97, either in the context of CDK7/cyclin H or independent of these host factors, may contribute to the control of transcriptional activity in HCMV-infected cells [10,11,22,23]. To this end, we generated experimental conditions described previously, which included a transient knockout of cyclin H, specific inhibition of vCDK/pUL97 by maribavir (MBV), or inhibition of host CDK7 by LDC4297. Transcriptional profiling was performed via the use of materials derived from the HCMV infection of primary human fibroblasts (HFFs) under these differential conditions. To this end, a gene ontology analysis was performed to assess the differentially expressed genes and the transcription patterns provided by distinct conditional changes. The data demonstrated the importance of both vCDK/pUL97 and CDK7 for an enhanced level of transcriptional activity in HCMV-infected cells, which is considered a result of increased C-terminal domain (CTD) phosphorylation and the activation of host RNA polymerase (RNAP II). A structural modeling approach was applied to predict the location of pUL97 in the preinitiation complex (PIC) of RNAP II-mediated eukaryotic transcription. Novel aspects contributing to a refined understanding of HCMV-specific transcription are discussed. 

## 2. Results and Discussion

### 2.1. Assessing the Impact of Either Cyclin H KO or Inhibition of pUL97 or CDK7 on the Transcriptome of HCMV-Infected HFFs Using Differential RNA-Seq Analysis

**Figure 1:**
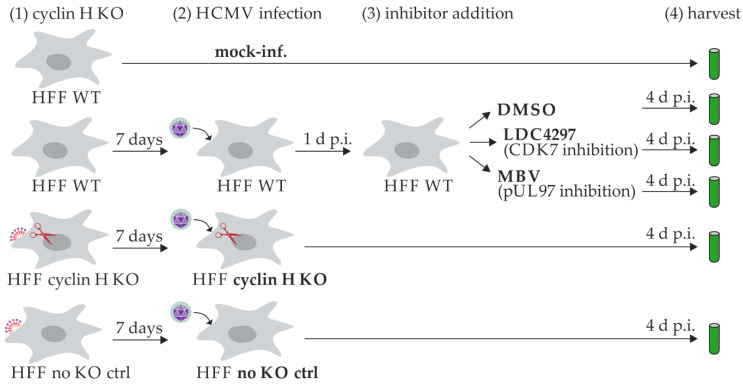
Schematic overview of the analyzed samples for the RNA-seq analysis. Individual steps of the procedure are indicated in the upper line: (1) HFFs were either treated under wild-type (WT) conditions or cyclin H knockout (KO) via lentiviral gene transduction. HFFs transduced with the CRISPR/Cas9 system without sgRNAs served as no KO controls. (2) Cells were infected with HCMV AD169 (MOI 0.1) or remained uninfected. (3) Inhibitors or solvent DMSO were added. (4) Cells were harvested and assayed in the RNA-seq procedure. Bold letters indicate the six analyzed treatment conditions, i.e., either uninfected control (mock-inf.), with (KO) or without cyclin H KO (no KO ctrl), or inhibitor treatment (DMSO, LDC4297, MBV).

An RNA-seq analysis was performed to gain specific insights into the impact of the proteins vCDK/pUL97, CDK7, and cyclin H on the transcriptome. Six different experimental conditions were analyzed in biological triplicates (Figure 1, see bold print): mock-infected HFFs and HCMV-infected HFFs that were either DMSO-treated, LDC4297-treated, MBV-treated, transient cyclin H KO, or no KO control.

**Figure 2:**
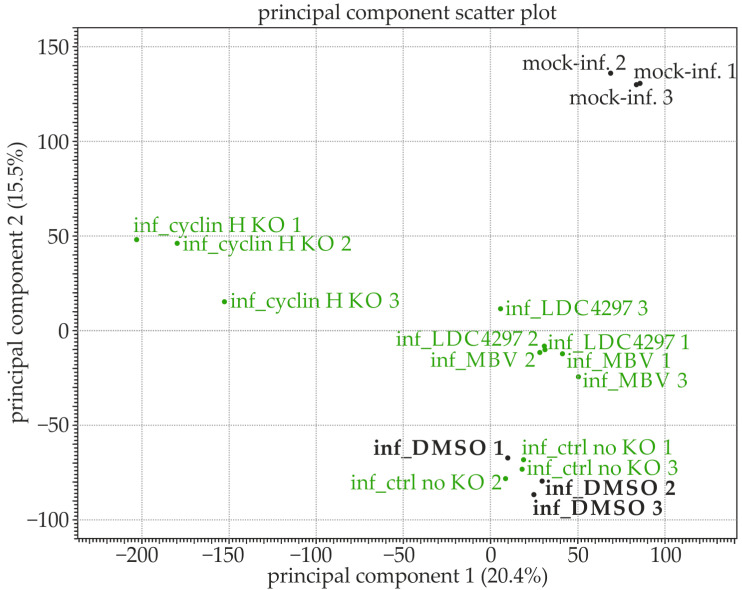
Principal component analysis (PCA): the individual biological triplicates are closely clustered, indicating consistency in their gene expression profiles. Each point on the scatter plot represents a sample, and its coordinates are determined by gene expression levels. Samples with similar gene expression profiles are positioned close together. The PCA plot displays the most significant patterns of variance within the high-dimensional dataset. Principal component 1 accounted for 20.4% of the total variance, capturing the primary pattern in the data. Principal component 2 represents the second most significant direction of variance and accounted for 15.5% of the total variance. Black indicates uninfected/mock control samples (non-bold) or DMSO-treated samples of HCMV infection (bold); green indicates samples of inhibitor treatment (MBV, LDC4297) or cyclin H knockout (KO).

A Western blot analysis confirmed that cyclin H was downregulated to 24% compared with the mock-infected control. Consistent with our previously published findings, cyclin H was significantly upregulated in all experimental conditions when compared with the mock infection (139% for inf_ctrl no KO, 169% for inf_DMSO, 170% for inf_MBV, and 134% for inf_LDC4297; Figure S1). A quality assessment of all samples was routinely performed. The representative quality report of one sample for the sequencing and gene alignment is available in the Supplementary Materials (Figures S2 and S3). The RNA-seq data demonstrated high quality across all conditions and was appropriate for differential gene analysis. A list containing all human and viral genes with the detected expression counts, annotations, and statistics of all samples can be found in Table S1. In the first step, the general gene expression levels of the individual samples were visualized using a principal component analysis (PCA, Figure 2). In this synoptic plot, the individual samples from the highly complex RNA-seq dataset are generally distinguished from each other based on the concept of variance. Variance describes the degree to which data points deviate from the average and can be used to measure the spread of the data. The plot displays the individual samples in which the *x*-axis represents principal component 1 (which captured 20.4% of the total variance) and the *y*-axis signifies principal component 2 (accounting for 15.5% of the second most significant variance direction). Proximity on the plot indicates similarity in gene expression between samples. Here, the biological replicates of matching samples clustered closely, indicating only slight differences within the same treatment group but significant differences between the various treatment groups. Notably, the mock-infected samples (Figure 2, top right corner) and the cyclin H KO samples (left side) differed to the greatest extent from the HCMV-infected samples, highlighting the substantial transcriptional impact exerted by both HCMV infection and cyclin H KO. When comparing LDC4297 and MBV treatments, both inhibitors showed close clustering, indicating only a minor degree of transcriptomic alterations induced by the two inhibitors. Furthermore, the DMSO-treated samples and the no KO ctrl samples displayed a high level of similarity by clustering together in the bottom right corner (Figure 2). In the subsequent differential analysis, all samples were evaluated in their deviation from the DMSO-treated samples.

### 2.2. Gene Ontology Analysis of Differentially Expressed Genes in Biological Pathways

Gene ontology (GO) analysis was employed to gain a deepened understanding of the functional implications of the various applied conditions. In this approach, differentially expressed (DE) genes were assigned to a set of standardized biological processes [24,25]. The biological processes affected by HCMV infection (i.e., in DMSO-treated samples) compared with mock-infected cells are listed in Table S2. The processes most severely affected by HCMV infection were identified as cell cycle, biosynthetic processes (i.e., the formation of substances), and catabolic processes (i.e., the breakdown of substances). These results are in line with previous studies, suggesting that HCMV infection induces a cell cycle arrest at the G1/S border, and thus, creates a highly metabolic environment termed pseudomitosis [26]. Notably, equal cell seeding densities were used for all conditions of these experimental settings, thereby ensuring a consistent starting point for all experimental groups. Thus, equal cell numbers across various treatments and conditions (also including cyclin H KO) were microscopically confirmed at the time points of transduction, infection, and harvest. Specific attention was paid to the transcriptomic changes resulting from cyclin H KO, MBV, or LDC4297 treatments using DMSO-treated samples as the control group (Table 1). Most of the biological processes affected by these treatments were also differentially expressed when comparing mock-infected samples with HCMV-infected DMSO controls. This indicates that processes correlating with activities of cyclin H, CDK7, and/or pUL97 refer to major transcriptomic alterations of HCMV infection. Of note, cyclin H KO resulted in additional changes that were not observed in any other condition (i.e., biological regulation, response to stimulus, developmental process, cellular component organization, and cellular process; Table 1). The pronounced transcriptomic changes observed in the cyclin H KO condition could be attributed to the KO timing. Cyclin H KO was initiated one week before infection to ensure low cyclin H protein levels at the time point of infection. This early KO experimental KO was methodologically essential due to the continued intracellular maintenance of cyclin H levels, which might otherwise have masked the effects of the KO during the infection. In comparison, the inhibitors MBV and LDC4297 were added at 1 d p.i. with concentrations given by the antiviral EC_50_ values. Interestingly, all conditions analyzed showed an impact on the regulation of the cell cycle (Table 1). Specifically, cyclin H KO manifested a change in 158 DE genes, LDC4297 changed 64 DE genes, and MBV changed 17 DE genes when compared with the DMSO control samples. These processes altered by LDC4297 also showed alteration by cyclin H KO, underscoring the crucial role of cyclin H for CDK7 activity. Interestingly, MBV exerted a notable effect on the DNA metabolic process, which is a process that remained unaffected by either cyclin H KO or LDC4297 treatment. This observation suggests that vCDK/pUL97 modifies cellular processes independently of cyclin H and CDK7, illustrating its multifaceted role in the transcriptional regulation of HCMV infection.

### 2.3. Deciphering Transcriptional Patterns Provided by the Distinct Conditions of HCMV Infection

**Figure 3:**
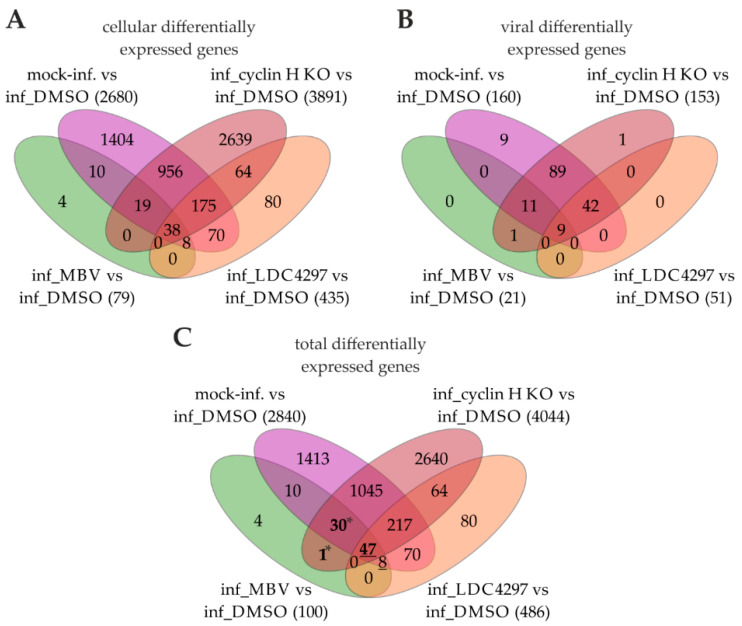
Venn-diagram-based data evaluation of differential gene expression in HCMV-infected HFFs under various conditions. Gene expression in DMSO-treated samples served as a reference and was compared with mock-infected, cyclin H KO, MBV-treated, and LDC4297-treated samples. The number of significantly differentially expressed (DE) genes (*p* ≤ 0.05) with at least a 1.5-fold change are listed. DE genes were categorized into cellular transcripts (**A**), viral transcripts (**B**), and a combination of both cellular and viral transcripts (**C**). Bold numbers indicate DE genes in MBV and cyclin H KO; underlined numbers indicate DE genes in MBV and LDC4297; asterisks indicate DE genes in MBV and cyclin H KO, but not in LDC4297.

Subsequently, the individual gene expressions in HCMV-infected DMSO control samples were compared with those of mock-infected cells, cyclin H KO, MBV, and LDC4297. Venn diagrams depict the number of DE genes that showed significant alteration, each with a minimum of a 1.5-fold change (Figure 3). These genes were then categorized into cellular genes (Figure 3A), viral genes (Figure 3B), and a combination of both cellular and viral genes (Figure 3C). HCMV infection, as represented by the DMSO-treated samples, caused significant changes in 2840 genes when compared with mock-infected samples, which is consistent with prior studies highlighting the effects of HCMV infection on host transcription [27]. Among these, 2680 were cellular genes and 160 were viral genes. Notably, cyclin H KO led to the most profound transcriptomic changes, affecting a total of 4044 genes (3891 of cellular origin and 153 of viral origin). CDK7 inhibition with LDC4297 resulted in 486 DE genes (51 of which were viral), while pUL97 inhibition by MBV resulted in 100 changes (including 21 viral DE genes). Surprisingly, both MBV and LDC4297 had a more substantial impact on cellular than on viral genes, affecting only a small fraction of the total 162 detected viral genes. This may underline the fact that the modulatory effect, which was exerted through vCDK/pUL97 and CDK7 activity, was more pronounced for the cellular transcriptional machinery than for viral gene regulation. In contrast, cyclin H KO affected 153 genes, which was the highest number of DE viral genes compared to MBV and LDC4297. This was likely due to the timing of the KO, which was initiated earlier than the addition of the inhibitors. Interestingly, cyclin H KO, MBV, and LDC4297 treatments affected similar genes. This may suggest that the CTD-specific phospho-activation of RNAP II, which was mediated through CDK7, cyclin H, and vCDK/pUL97, possibly favored some target transcripts. A total of 78 of the 100 DE genes altered by MBV were also impacted by cyclin H KO (Figure 3C, bold numbers). LDC4297 and MBV shared 55 overlapping DE genes (Figure 3C, underlined numbers); 31 genes were impacted by both cyclin H KO and MBV but were unchanged by the LDC4297 treatment (Figure 3C, numbers with asterisks).

In particular, the 31 DE genes that overlapped between MBV and cyclin H KO suggest that these genes might have been regulated through the pUL97–cyclin H interaction independently of CDK7 (Table 2). Among these genes, 12 were of viral origin, and the majority were involved in immune evasion. In MBV and cyclin H KO conditions, the expression of these HCMV transcripts decreased by 1.5- to 2.3-fold when compared with the DMSO control. This indicates a vCDK/pUL97 and cyclin H dependence of these transcripts. Interestingly, in this group of 31 DE genes, the MBV condition exerted an effect that was not exclusively on HCMV genes but also on functionally important host cell genes. These included genes involved in cell cycle regulation, DNA replication, and signal transduction. Notably, the mRNA levels of cyclin E, which is a protein involved in controlling the G1-S phase transition and known to accumulate in HCMV-infected cells [28], were found to be decreased upon cyclin H KO and MBV addition. Another interesting finding was that MBV and cyclin H KO inhibited the HCMV-induced upregulation of transcription repression factor BEN domain containing 3 (*BEND3*), which binds over 800 promoters, including that of CDK inhibitor p21 [29]. HCMV infection typically results in p21 downregulation and modulation of CDK activity [28]. Thus, a mechanistic link between the *BEND3* protein and pUL97 transcriptional activity appeared plausible. Principally, such aspects of pUL97 kinase functionality might be subject to distinct differences in protein expression by viral strains, as illustrated by Wang et al. [30]. To address the putative impact of viral strains on the transcription-related activity of pUL97, the two most affected transcripts identified by our transcriptomic analysis, namely, *CCNE1* and *BEND3*, were exemplarily reconsidered using an independent setting of RNA-specific RT-qPCR. To this end, three viral strains were assessed in parallel, i.e., HCMV AD169, Toledo, and Merlin, and the conditions of infection, as well as cyclin H KO, were identical to the procedures described before. The findings demonstrated that the infection with either of the three strains led to a statistically significant upregulation of the two mRNA levels of interest, and also here, a significant downregulation of these mRNAs by cyclin H KO could be detected (Figure S4). Thus, RT-qPCR independently confirmed the impact of cyclin H KO on the mRNA levels of *CCNE1* and *BEND3* for various HCMV strains, including the genetically intact strain Merlin (Figure S4).

### 2.4. Structural Analysis

**Figure 4:**
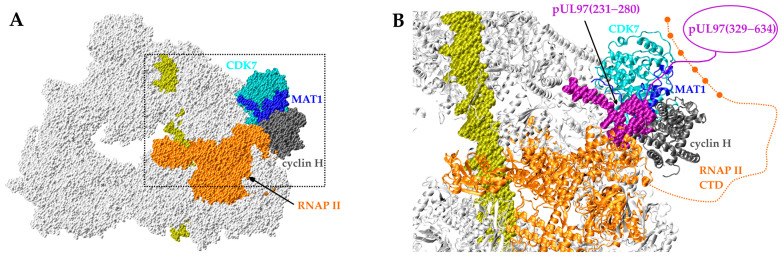
Predicted location of pUL97 in the preinitiation complex (PIC) of RNAP II-mediated eukaryotic transcription. (**A**) Structure of the RNAP II-specific PIC in white-space-filled presentation with the bound template DNA in yellow. The CDK7-MAT1-cyclin H unit, located at the periphery of the PIC, in association with RNAP II (1–1487) are shown in colors. The region in the dashed box is shown as an enlargement in panel (**B**). (**B**) The putative pUL97 binding site in the PIC (ribbon presentation of the PIC proteins). Note that the main cyclin-binding interface (IF2) of pUL97, comprising amino acids 231–280, is shown in the purple-space-filled presentation and the pUL97 (329–634) kinase domain is schematically outlined as a purple oval. The C-terminal domain (CTD) of RNAP II, which is not resolved in the experimental PIC structure, is shown as an orange dashed line. Filled orange spots indicate CTD phosphorylation sites located in spatial proximity of CDK7 and pUL97.

The results above demonstrate that CDK7 and vCDK/pUL97 are both functionally relevant for the transcriptional activation of RNAP II. Other studies have highlighted the importance of CDK7 for CTD phosphorylation in the context of HCMV infection [31,32]. Additionally, a recent study investigated the dynamics of the RNAP II preinitiation complex (PIC) in the context of HCMV infection [33]. This prompted us to further investigate the spatial arrangement of CDK7, pUL97, and RNAP II using a structure of the eukaryotic PIC that had been determined via electron microscopy (Figure 4A). This experimental structure contained CDK7, MAT1, and cyclin H, but lacked information about the putative position of pUL97. Our previous molecular modeling study suggested that the primary interaction motif of pUL97(231–280) used the same binding pocket as MAT1 for targeting the cyclin H–CDK7 complex [22]. The modeling indicated that this binding mode of pUL97(231–280) was also largely compatible with the PIC geometry (Figure 4B). This binding position would place the pUL97 kinase domain (residues 329–634) in close vicinity of CDK7 and the CTD of RNAP II. Thus, both CDK7 and pUL97 were in a position that should favor the interaction with RNAP II CTD for efficient phosphorylation. This model offers a structural explanation for the experimental finding that the stated regulatory interplay between CDK7 and vCDK/pUL97 may substantially contribute to RNAP II activation in HCMV-infected cells.

## 3. Materials and Methods

### 3.1. Cells, Viruses, and Antiviral Compounds

Primary human foreskin fibroblasts (HFFs, C0045C, Thermo Fisher Scientific, Waltham, MA, USA) were cultured in minimal essential medium (MEM, 21090055, Thermo Fisher Scientific) containing 10% FCS, 1× GlutaMAXTM (35050038, Thermo Fisher Scientific), and 10 g/mL gentamicin (22185.03 SERVA, Heidelberg, Germany). Cultured cells were maintained at 37 °C, 5% CO_2_, and 80% humidity. HCMV strains AD169, Toledo, and Merlin were used for infection at an MOI of 0.1. Virus inocula were replaced with fresh growth medium after incubation at 37 °C for 90 min. To enhance the HCMV Merlin and Toledo infection rates, we included a 30 min centrifugation step at 2500 rpm (2300× *g*) at room temperature during the 90 min inoculation period. The CDK7-specific inhibitor LDC4297 was obtained from Lead Discovery Center GmbH, Dortmund, Germany. pUL97-specific inhibitor MBV was obtained from MedChemExpress, Monmouth Junction, NJ, USA.

### 3.2. Total Transcriptome RNA-Seq Analysis

Early passage numbers (<10) of HFFs were seeded in 6-well plates. To analyze the specific effects of cyclin H KO, 3 wells were transduced the following day with the CRISPR/Cas9 system using sgRNAs A and B against cyclin H that were published before [11]. HFFs transduced with the CRISPR/Cas9 system lacking sgRNAs served as negative controls. The medium was replaced 1 day after transduction. Transduced and WT HFFs were infected with HFF AD169 6 days after transduction or remained mock-infected. Optionally, infected cells were treated with the half-maximal HCMV inhibitory (EC_50_) concentrations of pUL97 inhibitor MBV (0.35 µM), CDK7 inhibitor LDC4297 (0.09 µM), or equal amounts of DMSO. In total, 6 different samples were analyzed in biological triplicates: mock-infected HFFs, HCMV-infected HFFs DMSO-treated, HCMV-infected HFFs LDC4297-treated, HCMV-infected HFFs MBV-treated, HCMV-infected transient cyclin H KO HFFs, and HCMV-infected no KO control HFFs. HFFs were harvested 4 d p.i. and Western blot analysis was performed to validate the cyclin H KO. To this end, one-sixth of the total cells used for the RNA-seq analysis were lysed as described previously [10]. The biological triplicates of each condition were pooled for the Western blot analysis. Total RNA was extracted from the remaining cells using the Quick-RNA MiniPrep Plus Kit (R1057, Zymo Research Europe GmbH, Freiburg, Germany). An RNA library was prepared according to the manufacturer’s protocol using the NEBNext^®^ Ultra II Directional RNA Library Prep Kit for Illumina^®^ (E7760S, New England Biolabs GmbH, Frankfurt am Main, Germany), together with the NEBNext^®^ poly(A) mRNA Magnetic Isolation Module for mRNA enrichment (E7490S, New England Biolabs GmbH) and the NEBNext^®^ Multiplex Oligos for Illumina^®^ (E7780S, New England Biolabs GmbH). AMPure XP beads (Beckman Coulter GmbH, Krefeld, Germany) were used for the purification steps. A total of 1 µg of total RNA was used for the library preparation. The 18 libraries were sequenced on the Illumina NovaSeq6000 platform (Illumina Inc., San Diego, CA, USA) at 2 × 108 bp, resulting in 16,386,330 to 22,491,076 read pairs per sample. Samples were analyzed using the CLC Workbench (QIAGEN GmbH, Hilden, Germany) version 23.04 and its implemented Biomedical Workflow Identify and Annotate Differentially Expressed Genes and Pathway. First, quality reports were generated to monitor the integrity of the raw sequencing data. Then, low-quality bases and adapter sequences were removed using the built-in trimming tool. Reads were mapped to a combined reference genome comprising the human genome (GRCh38) and the HCMV strain Merlin (Accession NC_006273) genome using a HISAT2-based alignment tool. Gene expression levels were counted with a set Phred quality score of at least 30 (corresponding to an error probability of 0.1%). The raw counts were normalized using a trimmed mean of M-values (TMM). A generalized linear model (GLM) and a Wald test for statistical analysis were employed to examine differential gene expression between various treatments. Genes that exhibited at least a 1.5-fold change and a false discovery rate (FDR) *p*-value of less than 0.05 were considered differentially expressed. These genes were further analyzed for affected biological pathways using the Gene Ontology (GO) platform in CLC Workbench with default parameters. For the deposition of the entire data set of the transcriptomic RNA-seq analysis, see BioProject ID PRJNA1048317.

### 3.3. Structural Analysis

The structural features underlying the effect of pUL97 on RNAP II-mediated transcription were investigated using the structure of the eukaryotic preinitiation complex, including CDK7, MAT1, and cyclin H (PDB:7EGB) [34]. The binding site of pUL97(231–280) was adopted from the previous report by Schütz et al., 2023 [22]. Structural analysis and visualization were performed with VMD [35].

### 3.4. Reverse Transcriptase Quantitative PCR (RT-qPCR)

HFFs were infected with HCMV Merlin and Toledo at an MOI of 0.1 in 12-well plates or remained mock-infected. Cells underwent treatment with either DMSO or were subjected to cyclin H KO, as described in Section 3.2. Total RNA was extracted using the Quick-RNA MiniPrep Plus Kit (R1057, Zymo Research Europe GmbH). Additionally, total RNA extracted from AD169-infected cells for RNA-seq analysis was utilized. RNA concentrations were measured using the Qubit^®^ RNA Broad Range Kit (Thermo Fisher Scientific) and 50 ng of total RNA was used for the analysis. Relative mRNA levels of *CCNE1* and *BEND3* were determined via one-step RT-qPCR using the LunaScript RT SuperMix Kit (E3010, New England Biolabs GmbH) with specific primers and probes as follows:

CCNE1 forward 5′ AGGAAGAGGAAGGCAAACGTG;
CCNE1 reverse 5′ AATAATCCGAGGCTTGCACG;
CCNE1 probe 5′ CAGCCTTGGGACAATAATGC (5′ FAM and 3′ TAMRA);
BEND3 forward 5′ GATGCTGCTCTGGACTGCTC;
BEND3 reverse 5′ ATGCCTGCTAGGAGAGCCTC;
BEND3 probe 5′ CTGCAGGACTCCAGCAAACG (5′ FAM and 3′ TAMRA).

The obtained values were normalized to the DMSO controls.

## 4. Conclusions

In essence, the current RNA-seq analysis aligned with the previous findings of our group, strongly suggesting a functional interaction between cyclin H, CDK7, and vCDK/pUL97 [11,21,22], which potentially regulates various aspects of gene transcription. The main findings of the present RNA-seq analysis were as follows: (i) all chosen conditions of kinase inhibition or cyclin H KO showed a substantial change in transcriptional patterns in HCMV-infected cells; (ii) in quantitative terms, the cyclin H KO exerted a more drastic transcriptional change than CDK7 or vCDK/pUL97 inhibition; (iii) according to the GO data, those altered processes referring to cell cycle regulation were the only group found associated with all relevant conditional changes of HCMV infection, i.e., CDK7, vCDK/pUL97 inhibition, or cyclin H KO; (iv) the identified altered CDK7-specific processes (LDC4297) were found in an overlap with those detected for cyclin H KO; (v) however, within the altered vCDK/pUL97-specific processes (MBV), particularly the DNA metabolic processes, were not paralleled by cyclin H KO or CDK7 inhibition; (vi) concerning the regulation of individual transcripts, distinctly different patterns were detected, including one pattern in which the indicated transcripts were broadly influenced by CDK7, cyclin H, and pUL97, or patterns that indicated transcripts showing an exclusive regulatory linkage to cyclin H and pUL97, or others; and (vii) a bioinformatic model provided a structural rationale for the experimental observation that both CDK7 and vCDK/pUL97 have an impact on RNAP II activation in HCMV-infected cells. Combined, the transcriptomic analysis provided the first detailed evidence for the functional relevance of vCDK/pUL97 for general transcription in HCMV-infected primary human fibroblasts.

## Figures and Tables

**Table 1:** Differential expression of biological pathways across various conditions in HCMV-infected HFFs ^#^.

Biological Process (Accession Number) ^&^	DMSO-Treated Compared to					
	Cyclin H KO		LDC4297-Treated		MBV-Treated	
	DE Genes	Bonferroni *p*-Value	DE Genes	Bonferroni *p*-Value	DE Genes	Bonferroni *p*-Value
Cell cycle (0007049)	158	8.5 × 10^−11^	64	0 *	17	1.1 × 10^−9^
➢ Cell cycle process (0022402)	36	1.7 × 10^−4^	21	1.8 × 10^−14^		
➢ Chromosome segregation (0007059)	32	0.012	18	9.2 × 10^−11^		
➢ Mitosis (0007067)	36	1.7 × 10^−4^	21	1.8 × 10^−14^		
➢ Mitotic cell cycle process (1903047)	36	1.7 × 10^−4^	21	1.8 × 10^−14^		
Organelle organization (0006996)						
➢ Organelle fission (0048285)	36	1.7 × 10^−4^	21	1.8 × 10^−14^		
➢ Nuclear division (0000280)	36	1.7 × 10^−4^	21	1.8 × 10^−14^		
Catabolic process (0009056)						
➢ Heterocycle metabolic process (0046483)					11	0.010
➢ Cellular aromatic compound metabolic process (0006725)					11	0.010
➢ Organic cyclic compound metabolic process (1901360)					11	0.010
➢ Nucleobase-containing compound metabolic process (0006139)					11	0.010
➢ DNA metabolic process (0006259)					11	1.3 × 10^−4^
Biological regulation (0065007)	494	3.2 × 10^−8^				
➢ Regulation of biological process (0050789)	454	1.2 × 10^−8^				
➢ Regulation of cellular process (0050794)	454	1.2 × 10^−8^				
➢ Signal transduction (0007165)	454	1.2 × 10^−8^				
Response to stimulus (0050896)	361	4.8 × 10^−9^				
➢ Response to stress (0006950)	361	4.8 × 10^−9^				
Developmental process (0032502)	354	3.7 × 10^−4^				
➢ Anatomical structure development (0048856)	290	2.5 × 10^−4^				
➢ Multicellular organism development (0007275)	58	0.012				
➢ Embryo development (0009790)	58	0.012				
➢ Cell differentiation (0030154)	214	0.045				
Immune system process (0002376)	205	4.5 × 10^−5^				
Locomotion (0040011)	126	3.8 × 10^−4^				
Cellular component organization (0071840)						
➢ Extracellular structure organization (0043062)	60	4.3 × 10^−5^				
➢ Extracellular matrix organization (0030198)	60	4.3 × 10^−5^				
➢ Protein complex assembly (0065003)	92	0.042				
➢ Protein complex subunit organization (0071822)	92	0.042				
Cellular process (0009987)						
➢ Cell adhesion (0007155)	91	4.7 × 10^−4^				
➢ Cell motility (0048870)	88	6.7 × 10^−4^				
➢ Cell death (0008219)	106	0.023				
➢ Cell proliferation (0008283)	67	0.015				

^#^ This table lists biological pathways that were significantly and differentially affected when comparing DMSO-treated samples with cyclin H KO samples, LDC4297-treated samples, and MBV-treated samples. For each biological process, the number of differentially expressed genes (DE genes) and their corresponding Bonferroni-corrected *p*-values are provided. Significance is defined as *p* ≤ 0.05, as determined using Fisher’s test followed by post hoc Bonferroni correction. ^&^ Grey shading indicates broader categories of biological processes. Arrowheads indicate sub-processes within broader categories. Indented processes represent sub-processes of the directly preceding, less-indented process. The official Gene Ontology (GO) definitions of the biological processes can be retrieved from the amiGO2 platform using the respective accession numbers (https://amigo.geneontology.org/amigo accessed on 28 September 2023). All relevant definitions are also given in Table S3. * A *p*-value of 0 is defined by the software as a minimally small *p*-value below cut-off.

**Table 2:** Differentially expressed genes that were exclusively affected by cyclin H KO and MBV but not by LDC4297 treatment ^#^.

Differentially Expressed Gene, Protein Name ^&^	DMSO-Treated Compared with					
	MBV		Cyclin H KO		Mock-Inf.	
	Fold Change	FDR *p*-Value	Fold Change	FDR *p*-Value	Fold Change	FDR *p*-Value
Viral proteins						
*UL74*, envelope glycoprotein O (virus entry)	2.3 (−)	2.2 × 10^−18^	1.8 (−)	4.8 × 10^−10^	778.1 (+)	3.2 × 10^−22^
*US28*, envelope protein US28 (GPCR signaling)	1.9 (−)	1.9 × 10^−7^	2.0 (−)	1.4 × 10^−11^	2913.5 (+)	1.5 × 10^−57^
*UL18*, membrane glycoprotein UL18 (immune evasion)	1.9 (−)	1.5 × 10^−3^	2.4 (−)	1.3 × 10^−7^	1114.6 (+)	2.3 × 10^−4^
*US8*, membrane glycoprotein US8 (immune evasion)	1.7 (−)	1.6 × 10^−11^	2.4 (−)	3.1 × 10^−34^	2785.9 (+)	2.0 × 10^−18^
*US18*, membrane protein US18 (immune evasion)	1.6 (−)	5.6 × 10^−12^	2.4 (−)	2.8 × 10^−41^	1586.4 (+)	4.8 × 10^−170^
*UL78*, envelope protein UL78 (GPCR-like signaling)	1.6 (−)	1.9 × 10^−9^	2.7 (−)	1.8 × 10^−51^	1593.9 (+)	1.3 × 10^−170^
*UL72*, deoxyuridine triphosphatase UL72 (nucleotide metabolism)	1.5 (−)	3.2 × 10^−10^	2.0 (−)	6.0 × 10^−29^	1215.6 (+)	2.7 × 10^−57^
*UL114*, uracil-DNA glycosylase UL114 (DNA repair)	1.5 (−)	1.3 × 10^−7^	2.4 (−)	7.8 × 10^−36^	1858.4 (+)	1.7 × 10^−28^
*UL133*, protein UL133 (establishment of latency)	1.5 (−)	1.3 × 10^−3^	2.6 (−)	3.6 × 10^−18^	1789.6 (+)	6.9 × 10^−5^
*RL6*, protein RL6 (establishment of latency)	1.5 (−)	1.7 × 10^−3^	3.2 (−)	4.4 × 10^−28^	1136.3 (+)	1.5 × 10^−14^
*RL11*, membrane glycoprotein RL11 (immune evasion)	1.5 (−)	1.9 × 10^−2^	2.5 (−)	7.7 × 10^−14^	874.9 (+)	6.8 × 10^−105^
*UL40*, membrane glycoprotein UL40 (immune evasion)	12.6 (+)	4.4 × 10^−2^	36.2 (+)	5.6 × 10^−5^	13.2 (+)	5.2 × 10^−2^
Human proteins (cell cycle regulation)						
*CCNE1*, cyclin E1 (G1-S phase transition)	1.9 (−)	2.8 × 10^−6^	1.9 (−)	7.6 × 10^−8^	3.4 (+)	3.6 × 10^−20^
Human proteins (DNA replication, DNA repair, and transcription)						
*MCM2*, minichromosome maintenance complex component 2 (initiation of DNA replication)	1.7 (−)	6.7 × 10^−9^	1.6 (−)	2.4 × 10^−10^	2.6 (+)	1.1 × 10^−31^
*UBE2T*, ubiquitin conjugating enzyme E2 T (DNA repair)	1.5 (−)	2.0 × 10^−3^	1.9 (−)	3.4 × 10^−8^	3.0 (+)	6.3 × 10^−20^
*HELLS*, helicase, lymphoid specific (chromatin remodeling)	1.7 (−)	2.6 × 10^−4^	1.6 (−)	2.5 × 10^−4^	2.9 (+)	3.7 × 10^−17^
*GINS2*, GINS complex subunit 2 (DNA replication)	1.6 (−)	1.1 × 10^−2^	1.8 (−)	9.3 × 10^−5^	2.4 (+)	3.1 × 10^−9^
*BEND3*, BEN domain containing 3 (histone modification, transcriptional repressor)	1.6 (−)	2.1 × 10^−2^	2.3 (−)	3.0 × 10^−8^	4.3 (+)	2.5 × 10^−18^
Human proteins (signal transduction)						
*PASK*, PAS domain containing Ser/Thr kinase (energy homeostasis)	1.7 (−)	4.4 × 10^−2^	1.7 (−)	5.3 × 10^−3^	2.4 (+)	4.6 × 10^−6^
*NETO2*, neuropilin and tolloid like 2 (neurological functioning)	1.7 (−)	1.9 × 10^−7^	1.9 (−)	6.0 × 10^−12^	4.0 (+)	3.2 × 10^−40^
*RAB3IP*, RAB3A interacting protein (exocytosis and secretion)	1.6 (−)	4.4 × 10^−2^	1.7 (−)	1.4 × 10^−3^	5.2 (+)	4.3 × 10^−17^
Human proteins (membrane proteins)						
*ULBP2*, UL16 binding protein 2 (immune response)	1.7 (−)	8.6 × 10^−3^	1.6 (−)	4.0 × 10^−3^	5.3 (+)	1.1 × 10^−19^
*CLCA2*, chloride channel accessory 2 (ion transport)	1.7 (+)	2.5 × 10^−2^	24.6 (+)	2.7 × 10^−115^	1.8 (−)	6.8 × 10^−4^
Human proteins (cellular structure)						
*TTLL7*, tubulin tyrosine ligase like 7 (tubulin polyglutamylation)	1.5 (−)	1.1 × 10^−2^	1.8 (−)	2.6 × 10^−6^	1.8 (+)	3.7 × 10^−6^
Human proteins (extracellular components)						
*ASPN*, asporin (extracellular matrix formation)	1.8 (+)	2.7 × 10^−2^	2.0 (+)	3.6 × 10^−4^	2.9 (−)	2.4 × 10^−8^
Human proteins (cellular organelles)						
*BRI3BP*, BRI3 binding protein (mitochondria viability)	1.7 (−)	8.9 × 10^−5^	1.6 (−)	3.9 × 10^−5^	2.6 (+)	6.1 × 10^−16^
Human proteins (pseudogenes)						
*PPIAP22*, peptidylprolyl isomerase A pseudogene 22	1.5 (+)	2.1 × 10^−3^	-2.2 (+)	7.5 × 10^−15^	−1.5 (−)	1.4 × 10^−4^
*PDE4DIP*, phosphodiesterase 4D interacting protein pseudogene 2	1.8 (+)	1.3 × 10^−2^	-2.1 (+)	5.0 × 10^−5^	−1.8 (−)	2.0 × 10^−3^
*RPL14P1*, ribosomal protein L14 pseudogene 1	1.9 (+)	3.8 × 10^−8^	1.6 (+)	5.0 × 10^−5^	−2.1 (−)	4.1 × 10^−13^
*RPL15P3*, ribosomal protein L15 pseudogene 3	5.4 (+)	9.1 × 10^−3^	19.5 (+)	4.4 × 10^−10^	−6.3 (−)	3.1 × 10^−4^
*RPL41P1*, ribosomal protein L41 pseudogene 1	17.3 (+)	1.4 × 10^−2^	30.9 (+)	9.9 × 10^−5^	−13.5 (−)	5.2 × 10^−3^

^#^ This table lists significant differentially expressed (DE) genes when comparing HCMV infection of DMSO-treated samples with MBV-treated samples, cyclin H KO samples, and mock-infected cell samples. The fold change and their corresponding FDR *p*-values are provided (statistically significant *p* ≤ 0.05). Plus and minus symbols indicate whether the gene was up- or downregulated in the DMSO-treated samples compared with MBV, LDC4297, and mock-infected cells. ^&^ The names of the proteins translated from the DE genes investigated are given. A short description of protein function is provided in the brackets.

## Data Availability

The entire data set of the transcriptomic RNA-seq analysis has been deposited at BioProject ID PRJNA1048317.

## Supplementary Materials

The following supporting information can be downloaded at: https://www.mdpi.com/article/10.3390/ijms242417421/s1.
