# Supplementary material for: The Interactive Complex between Cytomegalovirus Kinase vCDK/pUL97 and Host Factors CDK7–Cyclin H Determines Individual Patterns of Transcription in Infected Cells
Source: Int J Mol Sci. 2023 Dec 13;24(24):17421. doi: 10.3390/ijms242417421 (PMC10744309; doi:10.3390/ijms242417421)
Supplement: Supplementary file 1 [file ijms-24-17421-s001.zip › Supplementary_Sch¿╣tz et al-revised.pdf]

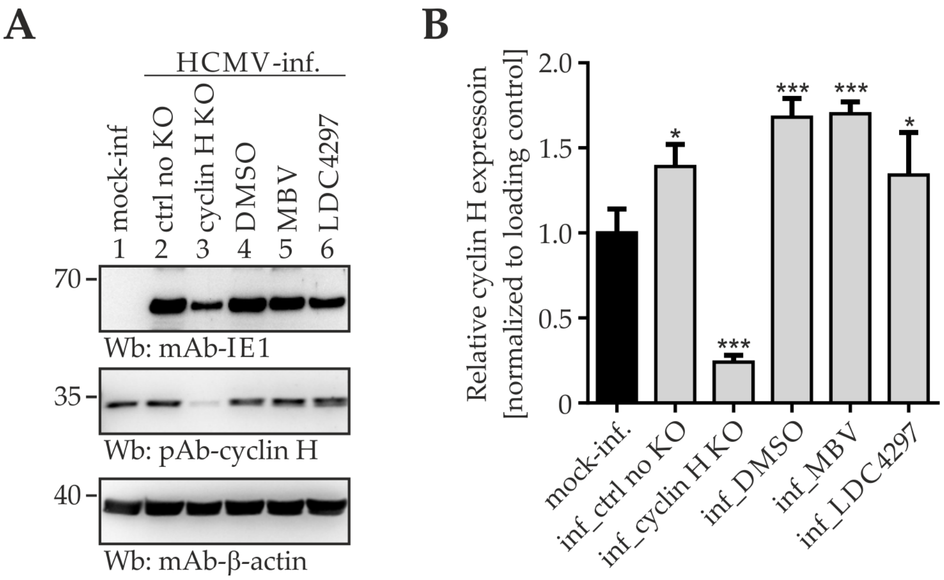

Figure S1. Confirmation of the successful cyclin H KO. HFFs were subjected to either wild-type conditions or had cyclin H knocked out (KO) through lentiviral gene transduction. HFFs that were transduced with the CRSISPR/Cas9 system without sgRNAs functioned as the no KO controls. After 7 days, the cells were either infected with HCMV AD169 (MOI 0.1) or left mock-infected. Inhibitors (MBV, LDC4297) or solvent DMSO were added 1 d p.i. The cells were harvested 4 days p.i. and 1/6 of the cells were analyzed by Western blot using the indicated antibodies **(A)**. Relative expression of cyclin H was determined quantitatively by evaluating the densitometry of three technical replicates of the samples and then normalizing to the loading control of β-actin **(B)**.

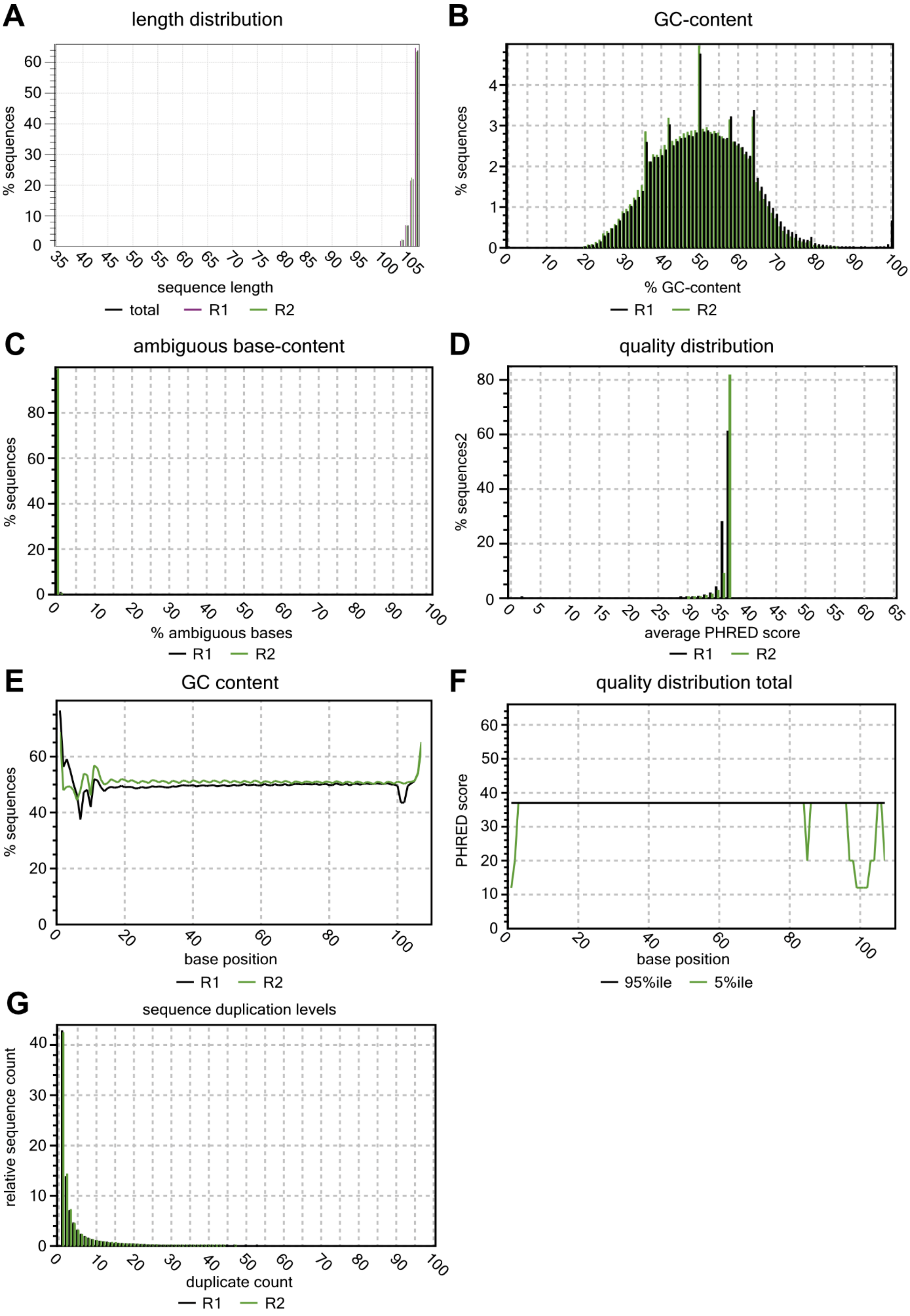
A
length distribution
% sequences
sequence length
total
R1
R2
B
GC-content
% sequences
% GC-content
R1
R2
C
ambiguous base-content
% sequences
% ambiguous bases
R1
R2
D
quality distribution
% sequences2
average PHRED score
R1
R2
E
GC content
% sequences
base position
R1
R2
F
quality distribution total
PHRED score
base position
95%ile
5%ile
G
sequence duplication levels
relative sequence count
duplicate count
R1
R2

Figure S2. Representative quality report of the RNA-seq data. This report displays the DMSO-treated sample 1 sequencing results based on 34,906,956 sequences. **(A)** The sequence length across all reads is depicted. **(B)** The GC content of a sequence is calculated as the number of GC bases compared to all bases. **(C)** The distribution of N-contents is shown. The N-content of a sequence is calculated as the number of ambiguous bases compared to all bases. Very low proportions close to zero indicate correct sequencing of most bases. **(D)** Distribution of average sequence quality scores. The quality of a sequence is calculated as the arithmetic mean of its base qualities. A Phred score above 30 (indicating a correctly sequenced base with a probability of 99.9%) indicates high sequencing quality. **(E)** Combined coverage of G- and C-bases. GC content should be relatively uniform after the first few bases and at the end. **(F)** Base-quality distribution along the base positions for total reads. The 95%ile represents the score below which 95% of the data falls, while the 5%ile represents the line below which 5% of the data falls for each position. The gap between the 95% and 5% lanes represents 90% of the data and gives insight into the variability of the quality score. **(G)** Duplication level distribution. Low levels are generally better, although some duplication may occur and will be corrected in the subsequent analysis. R1, forward reads; R2, reverse reads.

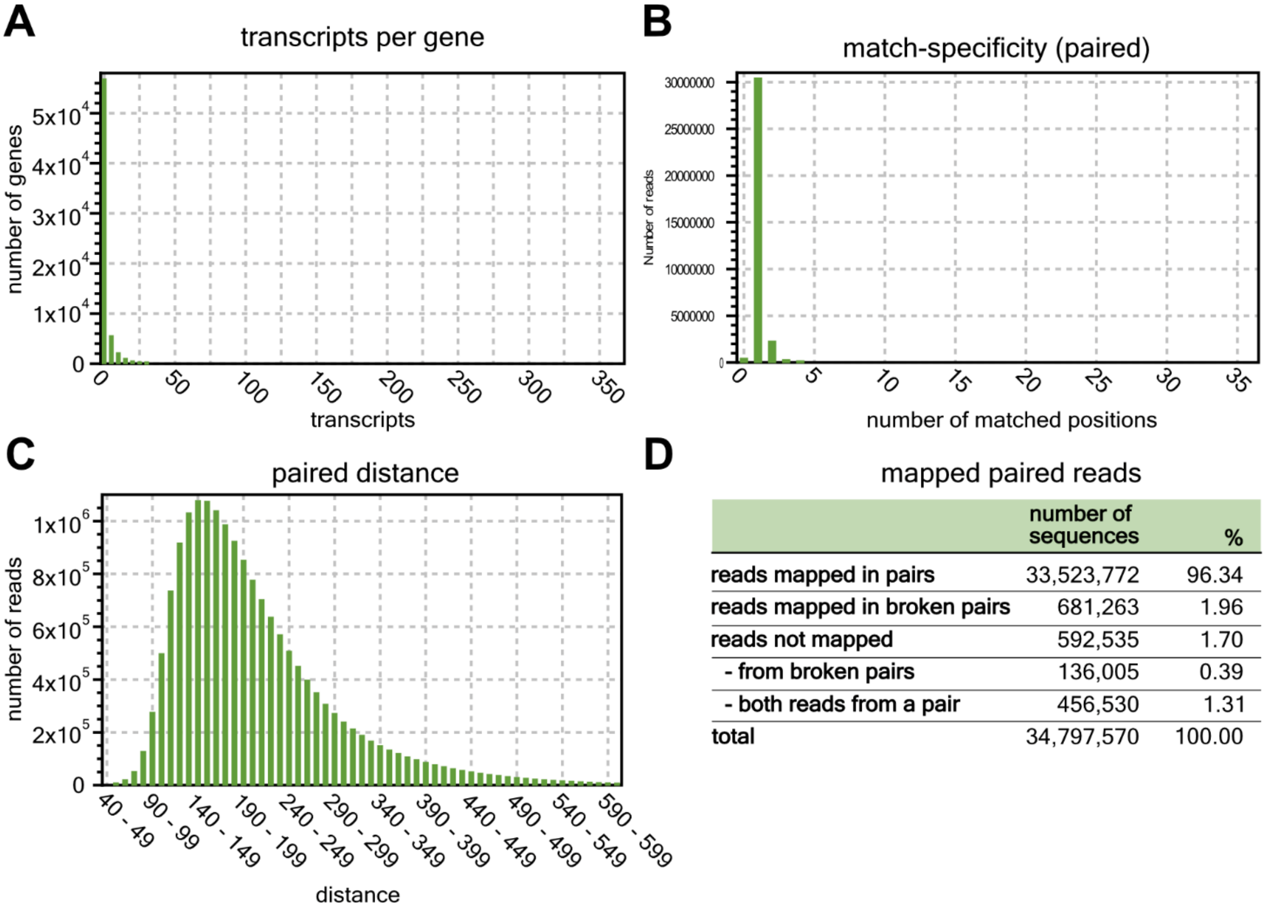

	number of sequences	%
reads mapped in pairs	33,523,772	96.34
reads mapped in broken pairs	681,263	1.96
reads not mapped	592,535	1.70
- from broken pairs	136,005	0.39
- both reads from a pair	456,530	1.31
total	34,797,570	100.00

Figure S3. RNA-seq report of the mapping. Shown is the report of DMSO-treated sample 1. **(A)** This graph illustrates the number of transcripts identified for each gene. **(B)** The graph indicates the number of times that reads could be mapped to the reference genome. A number of 1 means that the sequenced transcript could be unambiguously mapped to the reference genome. **(C)** The graph shows the distribution of paired-end distances, which is equivalent to the distribution of sequenced RNA fragment sizes. One single broad peak at the target fragment size indicates high quality. **(D)** The table shows the number and percentage of mapped reads. A high percentage >90% is considered as high quality.

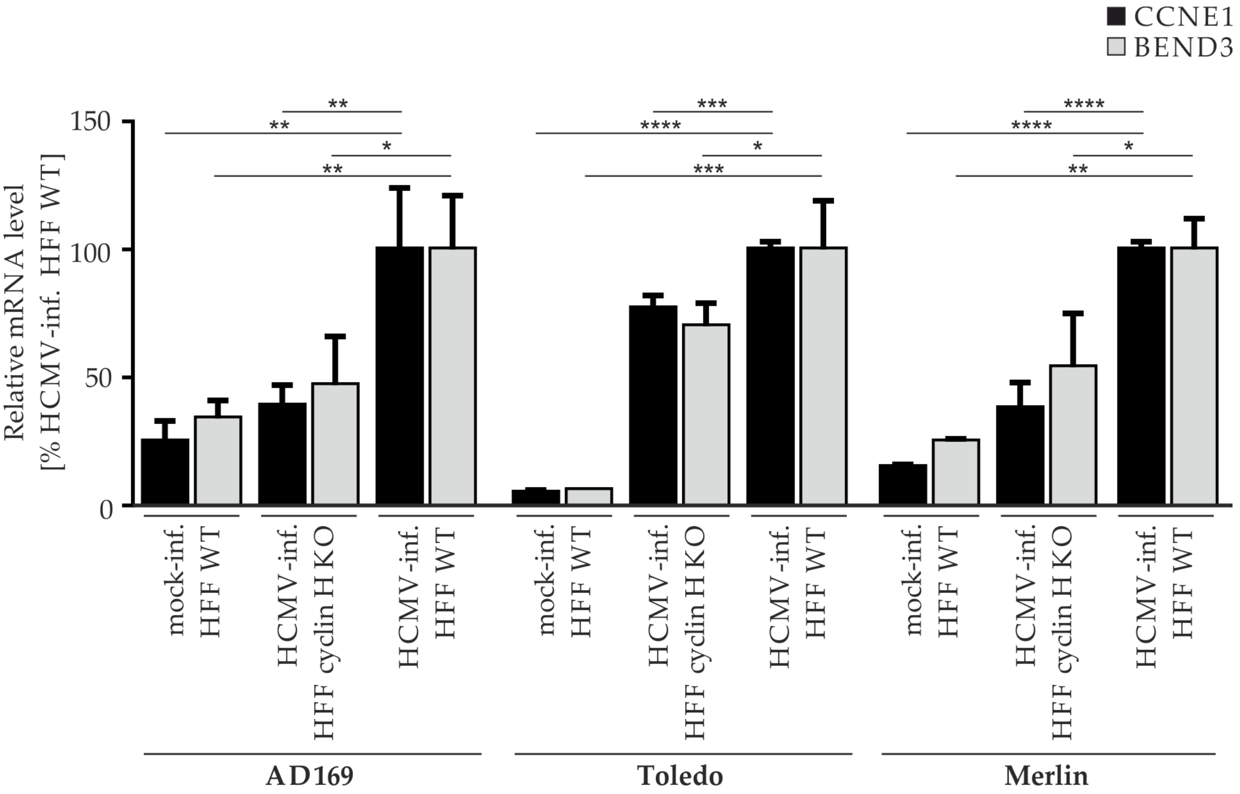

Figure S4. Confirmation of the impact of cyclin H KO on CCNE1 and BEND3 mRNA levels using RT-qPCR. HFFs were either mock-infected or infected with one of the three different strains of HCMV AD169, Toledo, or Merlin, at MOI of 0.1 to evaluate CCNE1 and BEND3 mRNA levels. Cells were treated with either DMSO or subjected to cyclin H KO under conditions noted for Figures 1-3. Cells were harvested 4 d p.i. and total RNA was extracted. The specific mRNA levels were measured with a one-step RT-qPCR and normalized against DMSO controls. Statistical analysis was performed using an ordinary one-way ANOVA test and post-hoc Dunnett correction compared to HCMV-inf. HFF WT *, $p<0.05$; **, $p<0.01$; ***, $p<0.001$; ****, $p<0.0001$.

Table S1. Listing of human and viral genes. The Table is available online as indicated in the manuscript.

Table S2. Differential expression of biological pathways in HCMV-infected versus mock-infected HFFs.[#]

Biological process (accession number)[&]	DE genes	Bonferroni p-value
**cell cycle (0007049)**	**130**	**0**
➢ cell cycle process (0022402)	30	3.6×10^{-5}
➢ chromosome segregation (0007059)	29	1.6×10^{-4}
➢ mitosis (0007067)	30	3.6×10^{-5}
➢ mitotic cell cycle process (1903047)	30	3.6×10^{-5}
**cellular component organization (0071840)**	**315**	**1.6×10^{-4}**
➢ extracellular structure organization (0043062)	40	7.5×10^{-3}
➢ extracellular matrix organization (0030198)	40	7.5×10^{-3}
➢ chromosome organization (0051276)	72	3.9×10^{-2}
➢ organelle organization		
➢ organelle fission (0048285)	30	3.6×10^{-5}
➢ nuclear division (0000280)	30	3.6×10^{-5}
**biosynthetic process (0009058)**	**219**	**3.5×10^{-3}**

➢ cellular biosynthetic process (0044249)	46	$1.1x10^{-3}$
➢ macromolecule biosynthetic process (0009059)	46	$1.1x10^{-3}$
➢ cellular nitrogen compound biosynthetic process (0044271)	46	$1.1x10^{-3}$
➢ organic substance biosynthetic process (1901576)	46	$1.1x10^{-3}$
➢ organonitrogen compound biosynthetic process (1901566)	46	$1.1x10^{-3}$
➢ peptide metabolic process (0006518)	46	$1.1x10^{-3}$
➢ peptide biosynthetic process (00430439	46	$1.1x10^{-3}$
➢ Translation (0006412)	46	$1.1x10^{-3}$
**nitrogen compound metabolic process (0006807)**	**251**	$\mathbf{4.2x10^{-2}}$
➢ amide metabolic process (0043603)	46	$1.1x10^{-3}$
➢ amide biosynthetic process (0043604)	46	$1.1x10^{-3}$
**response to stimulus (0050896)**	**240**	$\mathbf{6.5x10^{-5}}$
➢ response to stress (0006950)	240	$6.5x10^{-5}$
**catabolic process (0009056)**	**126**	$\mathbf{2.7x10^{-2}}$
➢ cellular catabolic process (0044248)	59	$2.7x10^{-10}$
➢ heterocycle metabolic process (0046483)	132	$3.7x10^{-8}$
➢ cellular aromatic compound metabolic process (0006725)	132	$3.7x10^{-8}$
➢ aromatic compound catabolic process (0019439)	59	$2.7x10^{-10}$
➢ organic substance catabolic process (1901575)	59	$2.7x10^{-10}$
➢ cellular nitrogen compound metabolic process (0034641)	248	$2.9x10^{-2}$
➢ cellular nitrogen compound catabolic process (0044270)	59	$2.7x10^{-10}$
➢ organic cyclic compound metabolic process (1901360)	132	$3.7x10^{-8}$
➢ organic cyclic compound catabolic process (1901361)	59	$2.7x10^{-10}$
➢ nucleobase-containing compound metabolic process (0006139)	132	$3.7x10^{-8}$
➢ nucleobase-containing compound catabolic process (0034655)	59	$2.7x10^{-10}$
➢ DNA metabolic process (0006259)	74	$1.2x10^{-5}$
**developmental process (0032502)**	**240**	$\mathbf{1.2x10^{-2}}$
➢ anatomical structure development (0048856)	203	$6.6x10^{-4}$
**localization (0051179)**		
organic substance transport (0071702)	49	$2.0x10^{-3}$
macromolecule localization (0033036)	49	$2.0x10^{-3}$
➢ protein localization (0008104)	49	$2.0x10^{-3}$
➢ establishment of protein localization (0045184)	49	$2.0x10^{-3}$
➢ protein transport (0015031)	49	$2.0x10^{-3}$
➢ intracellular protein transport (0006886)	49	$2.0x10^{-3}$
➢ protein targeting (0006605)	49	$2.0x10^{-3}$
**biological process involved in interspecies interaction between organisms (0044419)**	**58**	$\mathbf{2.6x10^{-3}}$
**cell proliferation (0008283)**	**52**	$\mathbf{3.8x10^{-3}}$

[#] The Table lists significant differentially expressed biological pathways when comparing DMSO-treated, HCMV-infected samples to mock-infected samples. The number of differentially expressed genes (DE genes) and their corresponding Bonferroni-corrected p-

values are provided for each biological process (statistically significant when $p \leq 0.05$ using Fisher's test and post-hock Bonferroni correction).

[&] Grey shading and bold writing indicate a more general classification of biological processes. The processes listed with arrowheads are respective sub-processes. More indented processes represent a subcategory of the above process. The official Gene Ontology (GO) definitions of the biological processes can be obtained from the amiGO2 platform using the respective accession numbers (https://amigo.geneontology.org/amigo).

Table S3. The official Gene Ontology definitions of the biological processes that were significantly affected by the cyclin H KO, MBV treatment, or LDC4297 treatment compared to DMSO.

**Biological process (accession number[&])**	**Definition**
**cell cycle process (0022402)**	The progression of biochemical and morphological phases and events that occur in a cell during successive cell replication or nuclear replication events.
chromosome segregation (0007059)	The process in which genetic material, in the form of chromosomes, is organized into specific structures and then physically separated and apportioned to two or more sets.
mitosis (0007067)	Progression through mitosis, the division of the eukaryotic cell nucleus to produce two daughter nuclei that, usually, contain the identical chromosome complement to their mother.
mitotic cell cycle process (1903047)	A process that is part of the mitotic cell cycle.
**organelle organization (0006996)**	A process that is carried out at the cellular level which results in the assembly, arrangement of constituent parts, or disassembly of an organelle within a cell.
organelle fission (0048285)	The creation of two or more organelles by division of one organelle.
nuclear division (0000280)	The division of a cell nucleus into two nuclei, with DNA and other nuclear contents distributed between the daughter nuclei.
**catabolic process (0009056)**	The chemical reactions and pathways resulting in the breakdown of substances, including the breakdown of carbon compounds with the liberation of energy for use by the cell or organism.
heterocycle metabolic process (0046483)	The chemical reactions and pathways involving heterocyclic compounds, those with a cyclic molecular structure and at least two different atoms in the ring (or rings).
cellular aromatic compound metabolic process (0006725)	The chemical reactions and pathways involving aromatic compounds, any organic compound characterized by one or more planar rings, each of which contains conjugated double bonds and delocalized pi electrons, as carried out by individual cells.
organic cyclic compound metabolic process (1901360)	The chemical reactions and pathways involving organic cyclic compound.
nucleobase-containing compound metabolic process (0006139)	Any cellular metabolic process involving nucleobases, nucleosides, nucleotides, and nucleic acids.
DNA metabolic process (0006259)	Any cellular metabolic process involving deoxyribonucleic acid.

**biological regulation (0065007)**	Any process that modulates a measurable attribute of any biological process, quality, or function.
regulation of biological process (0050789)	Any process that modulates the frequency, rate, or extent of a biological process. Biological processes are regulated by many means; examples include the control of gene expression, protein modification, or interaction with a protein or substrate molecule.
regulation of cellular process (0050794)	Any process that modulates the frequency, rate, or extent of a cellular process, any of those that are carried out at the cellular level, but are not necessarily restricted to a single cell.
signal transduction (0007165)	The cellular process in which a signal is conveyed to trigger a change in the activity or state of a cell.
**response to stimulus (0050896)**	Any process that results in a change in state or activity of a cell or an organism (in terms of movement, secretion, enzyme production, gene expression, etc.) as a result of a stimulus.
response to stress (0006950)	Any process that results in a change in state or activity of a cell or an organism (in terms of movement, secretion, enzyme production, gene expression, etc.) as a result of a disturbance in organismal or cellular homeostasis, usually, but not necessarily, exogenous (e.g. temperature, humidity, ionizing radiation).
**developmental process (0032502)**	A biological process whose specific outcome is the progression of an integrated living unit: an anatomical structure (which may be a subcellular structure, cell, tissue, or organ), or organism over time from an initial condition to a later condition.
anatomical structure development (0048856)	The biological process whose specific outcome is the progression of an anatomical structure from an initial condition to its mature state.
multicellular organism development (0007275)	The biological process whose specific outcome is the progression of a multicellular organism over time from an initial condition (e.g. a zygote or a young adult) to a later condition (e.g. a multicellular animal or an aged adult).
embryo development (0009790)	The process whose specific outcome is the progression of an embryo from its formation until the end of its embryonic life stage.
cell differentiation (0030154)	The cellular developmental process in which a relatively unspecialized cell, e.g. embryonic or regenerative cell, acquires specialized structural and/or functional features that characterize a specific cell.
**immune system process (0002376)**	Any process involved in the development or functioning of the immune system, an organismal system for calibrated responses to potential internal or invasive threats.
**locomotion (0040011)**	Self-propelled movement of a cell or organism from one location to another.
**cellular component organization (0016043)**	A process that results in the assembly, arrangement of constituent parts, or disassembly of a cellular component.
extracellular structure organization (0043062)	A process that is carried out at the cellular level which results in the assembly, arrangement of constituent parts, or disassembly of structures in the space external to the outermost structure of a cell.
extracellular matrix organization (0030198)	A process that is carried out at the cellular level which results in the assembly, arrangement of constituent parts, or disassembly of an extracellular matrix.
protein complex assembly (0065003)	The aggregation, arrangement, and bonding together of a set of macromolecules to form a protein-containing complex.
protein complex subunit organization (0071822)	Any process in which macromolecules aggregate, disaggregate, or are modified, resulting in the formation, disassembly, or alteration of a protein complex.

**cellular process (0009987)**	Any process that is carried out at the cellular level, but not necessarily restricted to a single cell. For example, cell communication occurs among more than one cell, but occurs at the cellular level.
cell adhesion (0007155)	The attachment of a cell, either to another cell or to an underlying substrate such as the extracellular matrix, via cell adhesion molecules.
cell motility (0048870)	Any process involved in the controlled self-propelled movement of a cell that results in translocation of the cell from one place to another.
cell death (0008219)	Any biological process that results in permanent cessation of all vital functions of a cell.
cell proliferation (0008283)	The multiplication or reproduction of cells, resulting in the expansion of a cell population.

[&] Grey shading and bold writing indicate broad categories of biological processes. The processes listed directly below are their respective subcategories. The official Gene Ontology (GO) definitions were obtained from the amiGO2 platform using the respective accession numbers (https://amigo.geneontology.org/amigo).
